# Staging laparoscopy for radiographically non-metastatic pancreatic ductal adenocarcinoma: cytology-driven upstaging and the SENDAI-SL risk-enrichment score

**DOI:** 10.1093/bjsopen/zrag176

**Published:** 2026-11-04

**Authors:** Daisuke Douchi, Shingo Yoshimachi, Naoki Rikiyama, Mika Ando, Yuichiro Umino, Hideaki Sato, Mitsuhiro Shimura, Koetsu Inoue, Masahiro Iseki, Shuichi Aoki, Takayuki Miura, Shimpei Maeda, Masaharu Ishida, Masamichi Mizuma, Atsushi Masamune, Michiaki Unno

**Affiliations:** Department of Surgery, Tohoku University Graduate School of Medicine, Sendai, Japan; Department of Surgery, Tohoku University Graduate School of Medicine, Sendai, Japan; Department of Surgery, Tohoku University Graduate School of Medicine, Sendai, Japan; Department of Surgery, Tohoku University Graduate School of Medicine, Sendai, Japan; Department of Surgery, Tohoku University Graduate School of Medicine, Sendai, Japan; Department of Surgery, Tohoku University Graduate School of Medicine, Sendai, Japan; Department of Surgery, Tohoku University Graduate School of Medicine, Sendai, Japan; Department of Surgery, Tohoku University Graduate School of Medicine, Sendai, Japan; Department of Surgery, Tohoku University Graduate School of Medicine, Sendai, Japan; Department of Surgery, Tohoku University Graduate School of Medicine, Sendai, Japan; Department of Surgery, Tohoku University Graduate School of Medicine, Sendai, Japan; Department of Surgery, Tohoku University Graduate School of Medicine, Sendai, Japan; Department of Surgery, Tohoku University Graduate School of Medicine, Sendai, Japan; Department of Surgery, Tohoku University Graduate School of Medicine, Sendai, Japan; Division of Gastroenterology, Tohoku University Graduate School of Medicine, Sendai, Japan; Department of Surgery, Tohoku University Graduate School of Medicine, Sendai, Japan

**Keywords:** peritoneal lavage cytology, occult metastasis, CA19-9, patient selection, resectability

## Abstract

**Background:**

Cross-sectional imaging may miss occult metastatic disease in pancreatic ductal adenocarcinoma and cannot detect positive peritoneal lavage cytology. This study quantified the yield of staging laparoscopy (SL) with routine lavage cytology and developed the SENDAI-SL score for risk-based prioritization.

**Methods:**

Consecutive patients with radiographically non-metastatic pancreatic ductal adenocarcinoma who underwent SL at one centre from January 2013 to June 2025 were analysed retrospectively. SL positivity was defined as liver metastasis, macroscopic peritoneal metastasis, and/or positive cytology. Multivariable logistic regression identified predictors; internal validation used 2000 bootstrap resamples.

**Results:**

Of 504 patients, 141 (28.0%) had positive SL. Positivity rates were 21.3%, 16.0%, 32.3%, and 41.7% in resectable, borderline resectable with portal vein involvement, borderline resectable with arterial involvement, and unresectable locally advanced disease, respectively. Gross liver or peritoneal metastases were detected in 51 patients (10.1%); cytology was positive in 125 (24.8%) and the sole finding in 90 of 141 positive procedures (63.8%). Among 499 patients with quantifiable serum carbohydrate antigen 19-9, unresectable locally advanced disease, body and/or tail location, tumour size ≥ 37 mm, and serum carbohydrate antigen 19-9 ≥ 180 U/ml were independently associated with SL positivity. The model's area under the receiver operating characteristic curve was 0.732 (optimism-corrected, 0.718). Score-specific positivity ranged from 8.6% to 83.3%. A score ≥ 2 prioritized 149 patients; 74 (49.7%) were positive, capturing 52.9% (74 of 140) of positives in the analysis cohort.

**Conclusions:**

SL upstaged 28.0% of patients, predominantly through cytology-only disease. The SENDAI-SL score may support prioritization when resources are limited but requires external validation and should not justify omitting SL.

## Teaser

In 504 consecutive patients with radiographically non-metastatic pancreatic ductal adenocarcinoma who underwent staging laparoscopy (SL) with routine peritoneal lavage cytology, 28.0% had occult metastatic findings—predominantly cytology-only disease (CY1 alone in 63.8% of positive laparoscopies)—with positivity rising from 21.3% in resectable to 41.7% in unresectable locally advanced disease. Four pre-SL decision-time factors (unresectable locally advanced status, body/tail location, tumour ≥ 37 mm, CA19-9 ≥ 180 U/ml) independently predicted positivity and were combined into a one-point-per-variable SENDAI-SL score that stratified observed positivity from 8.6% to 83.3% (optimism-corrected area under the curve 0.718). SL meaningfully upstaged more than one-quarter of patients, largely through cytology, and the SENDAI-SL score may help prioritize selective, risk-adapted SL when capacity is constrained.

## Introduction

Pancreatic ductal adenocarcinoma (PDAC) is characterized by early systemic and peritoneal dissemination, and even radiographically localized disease may harbour occult metastases. Cross-sectional imaging delineates local vascular anatomy and detects clinically evident metastases, but has limited sensitivity for low-volume peritoneal deposits and superficial liver metastases; it also cannot directly detect malignant cells in the peritoneal cavity^1,2^.

Staging laparoscopy (SL) addresses this gap by combining direct inspection of the peritoneal cavity and liver surface with peritoneal lavage cytology. However, its reported yield varies widely owing to differences in resectability status, routine use of cytology, timing relative to systemic therapy, and patient selection^3–7^. Therefore, the key clinical question is not whether SL detects occult metastases but which patients should undergo SL and at what point in the treatment pathway.

Positive peritoneal lavage cytology (CY1), defined by the presence of malignant cells in lavage fluid, is particularly important because it may be the sole indicator of occult dissemination in radiographically localized PDAC. Several Japanese series^8–10^ have shown that selected patients with CY1 disease can receive systemic therapy, achieve conversion to negative cytology (CY0), and subsequently undergo resection. Identifying CY1 before laparotomy may therefore redirect treatment sequencing without necessarily precluding subsequent surgery in selected responders.

This retrospective study evaluated 504 consecutive patients with radiographically non-metastatic PDAC who underwent SL with routine peritoneal lavage cytology at a high-volume tertiary centre. The study aimed to quantify the yield of SL across resectability strata, assess the contribution of cytology-only disease, identify simple pre-SL predictors of positivity, and develop a pragmatic SENDAI-SL score for risk-based prioritization rather than exclusion from SL.

## Methods

### Study design and patients

This retrospective single-centre cohort included consecutive patients with radiographically non-metastatic PDAC who underwent SL with peritoneal lavage cytology at Tohoku University Hospital between January 2013 and June 2025. Patients with distant metastases evident on imaging before SL were excluded. Diagnosis, resectability, treatment sequence, and SL indication were reviewed by a hepatopancreatobiliary multidisciplinary team. Diagnostic specimens were obtained by endoscopic ultrasound-guided fine-needle aspiration (EUS-FNA), endoscopic retrograde cholangiopancreatography (ERCP)-guided sampling, or other non-EUS-guided modalities according to tumour location, biliary anatomy, technical feasibility, and clinical judgement. Imaging at the initial clinical decision point was used to assign resectability, and pre-SL imaging was used to confirm the absence of radiographically evident distant metastasis. Positron emission tomography (PET) was performed in most patients as an adjunct to computed tomography (CT) and magnetic resonance imaging (MRI), although it was neither mandatory nor governed by a uniform protocol. PET was particularly considered when CT and/or MRI findings were equivocal or suspicious, tumour marker levels were unexpectedly high or discordant with imaging findings, extra-pancreatic disease was suspected, or the multidisciplinary team considered additional systemic staging clinically useful. The study was approved by the institutional review board (2025-1-239), which waived the requirement for individual informed consent, and reporting followed the STROBE statement^11^.

According to contemporaneous criteria used in routine practice^1,12,13^, resectability was classified as resectable, borderline resectable with portal vein involvement (BR-PV), borderline resectable with arterial involvement (BR-A), or unresectable locally advanced (UR-LA). Because imaging, neoadjuvant therapy, induction therapy, and institutional indications for SL evolved during the study period, resectability was analysed according to the category assigned at the initial clinical decision point. Patients were included irrespective of whether anticancer therapy had been initiated before the index laparoscopy, provided that no distant metastases were identified radiographically.

### SL and definitions

SL was performed under general anaesthesia using a standard laparoscopic approach, with systematic inspection of the diaphragmatic surfaces, liver surface, omentum, paracolic gutters, and pelvis. Suspicious lesions were biopsied whenever technically feasible^14^.

Before tumour manipulation, approximately 100 ml of sterile saline was instilled, and the recovered fluid was subjected to cytological examination. SL positivity was defined *a priori* as occult liver metastasis (H1), macroscopic peritoneal metastasis (P1), or CY1. Patients with none of these findings were classified as H0P0CY0.

In routine practice, CY1 without gross H1 and/or P1 disease was regarded as occult metastatic (M1-equivalent) disease for treatment sequencing; therefore, patients generally did not proceed directly to pancreatectomy but instead initiated or continued systemic chemotherapy. In selected patients, peritoneal lavage cytology was repeated at 2-month intervals via an abdominal port by instilling 100 ml of saline and recovering as much fluid as possible. Once cytological negativity had been confirmed on two consecutive assessments, imaging findings and tumour markers were reassessed. In patients without radiographic progression or new distant metastasis, with stable or decreased tumour marker levels, acceptable performance status, and technically resectable disease, repeat SL was performed selectively to reassess for residual occult metastatic disease, after which suitability for conversion surgery was determined by the multidisciplinary team. Because this was a retrospective real-world cohort, port-based cytological monitoring, subsequent reassessment, and repeat SL were performed selectively rather than according to a uniform prospective protocol.

### Pre-SL variables and outcomes

Clinical variables were recorded at the pre-SL decision point, defined as the latest assessment before the index-staging laparoscopy. Variables extracted from medical records included age, sex, calendar period of SL, resectability category, tumour location, tumour size, serum carbohydrate antigen 19-9 (CA19-9), carcinoembryonic antigen and cancer antigen 125 levels, PET use, maximum standardized uptake value (SUVmax) where available, method of tissue acquisition, and whether PDAC-directed treatment had been initiated before SL. Therefore, for chemotherapy-naïve patients, tumour size and tumour marker values represented initial pretreatment values, whereas for patients who had received anticancer treatment before SL, these variables represented post-treatment, pre-SL values. Resectability category was analysed according to the category assigned at the initial clinical decision point, because this category informed treatment sequencing and the indication for SL. Pre-SL treatment was defined as chemotherapy, radiotherapy, or chemoradiotherapy initiated before the index laparoscopy. Diagnostic procedures and supportive interventions, including EUS-FNA or ERCP-guided sampling, biliary drainage, antibiotics, analgesia, nutritional support, and other non-anticancer interventions, were excluded.

The primary outcome was SL positivity. Secondary outcomes included patterns of occult metastasis, subsequent curative-intent resection, performance of the simplified pre-SL risk-enrichment score, and exploratory associations between pre-SL treatment and positivity. Long-term survival after positive SL, disease-free survival (DFS) after subsequent resection, treatment sequence after positive SL, repeat or final cytology, and cytological conversion from CY1 to CY0 were outside the prespecified outcome framework. Therefore, available data for the SL-positive cohort were analysed *post hoc* as exploratory descriptive analyses.

### Statistical analysis

Continuous data are presented as the mean(standard deviation) or median (interquartile range (i.q.r.)), and categorical data as *n* (%). Between-group comparisons were performed using Welch's *t*-test or the Mann–Whitney *U* test for continuous variables and the χ^2^ or Fisher's exact test for categorical variables, as appropriate. Resectability-stratified comparisons of SL positivity, CY1, CY1-only disease, and gross H1 and/or P1 disease used the Fisher–Freeman–Halton exact test for 4 × 2 tables.

Logistic regression was used to examine factors associated with SL positivity. The clinically parsimonious multivariable model was fitted in the analysis cohort with quantifiable CA19-9 values (499) and included resectability category, tumour location, tumour size ≥ 37 mm, and CA19-9 ≥ 180 U/ml. The 37-mm tumour-size threshold was derived from the present cohort using preliminary receiver operating characteristic (ROC) analysis and was considered a data-derived risk-enrichment threshold requiring external validation. In patients treated before SL, this variable represented the most recent post-treatment, pre-SL imaging size and was not intended to represent viable tumour volume or true resectability after systemic therapy. CA19-9 was dichotomized at 180 U/ml using the ROC-derived threshold reported by Takadate *et al*.^14^ for predicting radiologically occult metastases in a previous staging-laparoscopy cohort. No new CA19-9 threshold was derived from the present cohort, nor were separate thresholds estimated by treatment status, because the post-treatment subgroup was smaller, clinically selected, and heterogeneous in treatment duration and response. CA19-9 kinetics were not incorporated because pretreatment and post-treatment values, timing of biliary drainage, treatment duration, and interval to SL were not uniformly available. CA19-9 was interpreted cautiously given the potential effects of cholestasis, biliary drainage, inflammation, and Lewis antigen status. The four model covariables were combined into an additive bedside score, assigning one point each for UR-LA disease, body and/or tail tumour location, tumour size ≥ 37 mm, and CA19-9 ≥ 180 U/ml.

The SENDAI-SL score combined the originally assigned resectability category and tumour location with tumour size and CA19-9 measured at the pre-SL decision point. In chemotherapy-naïve patients, tumour size and CA19-9 reflected baseline pretreatment values, whereas in patients who had received previous anticancer treatment they reflected the most recent post-treatment, pre-SL assessment. Only one tumour-size and one CA19-9 measurement from the same pre-SL decision point were used for each patient. Accordingly, the score was designed to estimate the probability of SL positivity at the time of SL decision-making, rather than to represent a fixed biological risk at initial diagnosis.

Model discrimination was summarized by the area under the ROC curve (AUC), and internal validation used 2000 bootstrap resamples to estimate the optimism-corrected AUC, Brier score, calibration intercept, and calibration slope, with calibration displayed using apparent and optimism-corrected locally weighted scatterplot smoothing curves. In 5 of 504 patients, CA19-9 was reported as < 2.0 U/ml, below the assay's lower limit of quantification. These five values below the limit of quantification were treated as non-quantifiable, and the corresponding patients were excluded from analyses involving CA19-9, including model development and score calculation; no imputation was performed.

The association between pre-SL treatment and SL positivity was explored using an additional adjusted model and stratified analyses. These analyses were considered exploratory because treatment before SL was not randomly assigned and patients who progressed before becoming eligible for SL were not represented in the SL cohort. All tests were two-sided, and statistical significance was set at *P* < 0.050.

In the *post hoc* analysis of SL-positive patients, overall survival (OS) was calculated from the index positive SL to death or last follow-up. In patients who subsequently underwent curative-intent resection, DFS was calculated from resection to recurrence or death, with censoring at last follow-up; analyses were limited to patients with valid postresection intervals. Survival was estimated using the Kaplan–Meier method, compared with the log-rank test, and summarized using the Mantel–Haenszel hazard ratio. Because subsequent resection depended on survival and disease control until patients became eligible for resection, these analyses were descriptive and did not include causal inference, landmark analysis, time-dependent modelling, or propensity adjustment.

## Results

### Cohort and pre-SL characteristics

*Table 1* summarizes the pre-SL characteristics recorded at the assessment closest to the index laparoscopy. Among 504 patients, the mean age was 68.9 years, 290 (57.5%) were men, and 145 (28.8%) had initiated anticancer treatment before SL. Tumours were classified as resectable in 263 patients (52.2%), BR-PV in 25 (5.0%), BR-A in 96 (19.0%), and UR-LA in 120 (23.8%); 208 (41.3%) were located in the body and/or tail. Compared with SL-negative patients, SL-positive patients more often had UR-LA disease (35.5% *versus* 19.3%), body and/or tail tumours (58.2% *versus* 34.7%), tumour size ≥ 37 mm (31.9% *versus* 11.6%), and CA19-9 ≥ 180 U/ml (43.6% *versus* 26.2%) (all *P* < 0.001), whereas age, sex, calendar period, and pre-SL treatment were similar between groups (*Table 1*).

**Table 1:** Pre-SL characteristics according to staging laparoscopy result

	Overall (*n* = 504)	SL-negative (*n* = 363)	SL-positive (*n* = 141)	P*
**Year of SL**				0.793
2013–2016	40 (7.9%)	27 (7.4%)	13 (9.2%)	
2017–2019	139 (27.6%)	100 (27.5%)	39 (27.7%)	
2020–2025	325 (64.5%)	236 (65.0%)	89 (63.1%)	
Age (years), mean(s.d.)	68.9(9.1)	68.9(9.1)	68.8(8.9)	0.887^a^
**Sex**				
Male	290 (57.5%)	209 (57.6%)	81 (57.4%)	0.979
Female	214 (42.5%)	154 (42.4%)	60 (42.6%)	
**Resectability**				< 0.001
R	263 (52.2%)	207 (57.0%)	56 (39.7%)	
BR	121 (24.0%)	86 (23.7%)	35 (24.8%)	
BR-PV	25 (5.0%)	21 (5.8%)	4 (2.8%)	
BR-A	96 (19.0%)	65 (17.9%)	31 (22.0%)	
UR-LA	120 (23.8%)	70 (19.3%)	50 (35.5%)	
**Tumour location**				< 0.001
Head	296 (58.7%)	237 (65.3%)	59 (41.8%)	
Body and/or tail	208 (41.3%)	126 (34.7%)	82 (58.2%)	
Tumour size (mm), median (i.q.r.)	25.0 (18.0–32.0)	24.0 (16.0–30.0)	30.0 (23.0–39.0)	< 0.001^b^
Tumour size ≥ 37 mm	87 (17.3%)	42 (11.6%)	45 (31.9%)	< 0.001
CA19-9 (U/ml), median (i.q.r.)†	64.5 (16.1–247.5)	48.9 (13.7–191.8)	129.8 (36.1–401.6)	< 0.001^b^
CA19-9 ≥ 180 U/ml†	155 (31.1%)	94 (26.2%)	61 (43.6%)	< 0.001
CEA (ng/ml), median (i.q.r.)	3.1 (2.0–5.1)	2.8 (2.0–4.8)	3.5 (2.3–6.3)	0.012^b^
CA125 (U/ml), median (i.q.r.)‡	13.5 (9.9–21.1)	12.7 (9.1–20.4)	16.1 (11.6–26.1)	0.005^b^
PET SUVmax, median (i.q.r.)§	5.6 (3.8–8.1)	5.5 (3.7–8.2)	6.0 (4.2–7.6)	0.441^b^
**Pre-SL treatment**	145 (28.8%)	106 (29.2%)	39 (27.7%)	0.731
Pre-SL treatment regimen				0.355
Gemcitabine plus nab-paclitaxel	67 (46.2%)	49 (46.2%)	18 (46.2%)	
Gemcitabine plus S-1	47 (32.4%)	37 (34.9%)	10 (25.6%)	
Modified FOLFIRINOX	11 (7.6%)	6 (5.7%)	5 (12.8%)	
Chemoradiation/proton beam therapy (± chemotherapy)	3 (2.1%)	3 (2.8%)	0 (0.0%)	
Multiple/other	17 (11.7%)	11 (10.4%)	6 (15.4%)	
**Preoperative tissue acquisition**				0.072
EUS-FNA	407 (80.8%)	286 (78.8%)	121 (85.8%)	
ERCP-guided sampling and other non–EUS-guided modalities	97 (19.2%)	77 (21.2%)	20 (14.2%)	

Values are *n* (%) unless otherwise stated. †CA19-9 analyses were restricted to patients with quantifiable values (499 overall, 359 SL-negative, 140 SL-positive); five values below the lower limit of quantification (< 2.0 U/ml) were excluded. ‡CA125 data were available for 291 patients. §PET SUVmax data were available for 423 patients. The resectability *P* value compares R, BR-PV, BR-A, and UR-LA. SL, staging laparoscopy; s.d., standard deviation; R, resectable; BR: borderline resectable, comprising BR-PV and BR-A; BR-PV, borderline resectable with portal vein involvement; BR-A, borderline resectable with arterial involvement; UR-LA, unresectable locally advanced; i.q.r., interquartile range; CA19-9, carbohydrate antigen 19-9; CEA, carcinoembryonic antigen; CA125, cancer antigen 125; PET, positron emission tomography; SUVmax: maximum standardized uptake value; EUS-FNA, endoscopic ultrasound-guided fine-needle aspiration; ERCP, endoscopic retrograde cholangiopancreatography. *Pearson’s χ² test unless otherwise indicated; ^a^Welch’s *t*-test; ^b^Mann–Whitney *U* test.

The subgroup patterns are summarized in *Fig. 1*. SL positivity was approximately twice as high for body and/or tail tumours as for head tumours (82 of 208, 39.4% *versus* 59 of 296, 19.9%), exceeded 50% in patients with tumours of at least 37 mm (45 of 87, 51.7%), and, in the analysis cohort with quantifiable CA19-9 values, was higher among patients with CA19-9 levels of at least 180 U/ml than among those below that threshold (61 of 155, 39.4% *versus* 79 of 344, 23.0%). In contrast, there was little difference in age and sex between patients who were SL-negative and SL-positive (*Table 1*).

**Figure 1:**
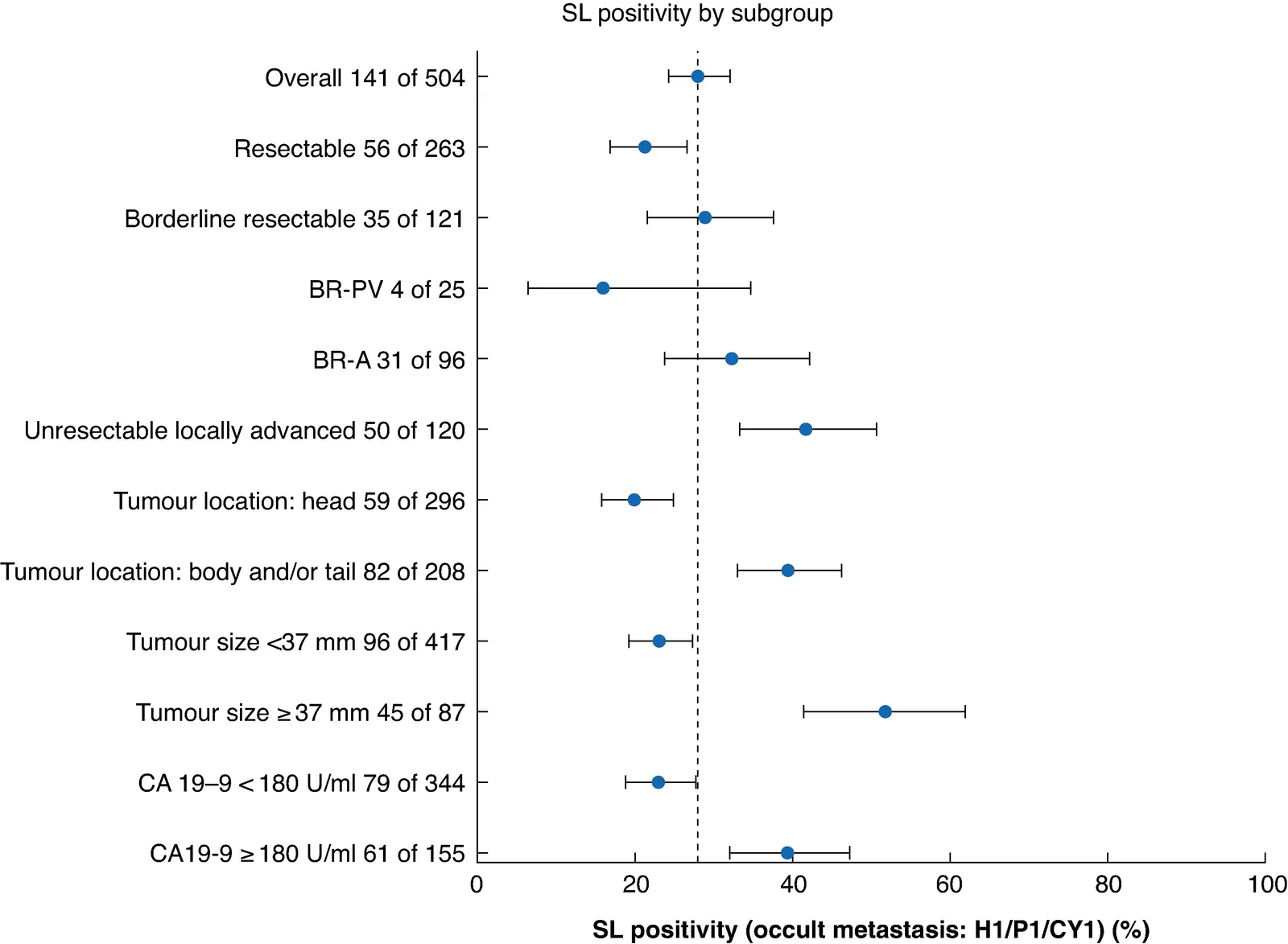
Observed staging laparoscopy positivity across clinically relevant pre-SL subgroups Dots represent proportions and horizontal lines represent 95% confidence intervals; the dashed vertical line indicates overall positivity (28.0%). Analyses of CA19-9 levels were restricted to patients with quantifiable values (499); five patients with values below the assay's lower limit of quantification (< 2.0 U/ml) were excluded. The global difference across detailed resectability categories was significant (*P* < 0.001). SL, staging laparoscopy; BR-PV, borderline resectable with portal vein involvement; BR-A: borderline resectable with arterial involvement; UR-LA, unresectable locally advanced; CA19-9, carbohydrate antigen 19-9; H1, liver metastasis; P1, macroscopic peritoneal metastasis; CY1, positive peritoneal cytology.

### Diagnostic yield and occult metastatic patterns

Overall, 141 of 504 patients had occult metastatic findings on SL, corresponding to a positivity rate of 28.0% (95% confidence interval (c.i.) 24.2 to 32.1). SL positivity differed significantly across resectability categories (global *P* < 0.001). Positivity rates were 21.3% (56 of 263) in resectable disease, 16.0% (4 of 25) in BR-PV, 32.3% (31 of 96) in BR-A, and 41.7% (50 of 120) in UR-LA disease (*Fig. 1* and *Table 2*). Combining BR-PV and BR-A yielded a positivity rate of 28.9% (35 of 121) for borderline resectable (BR) disease (*Fig. 1*). A resectability-stratified summary of occult metastatic patterns is provided in *Table S1*. The frequencies of overall SL positivity, CY1, and CY1-only disease differed significantly across the four resectability categories (all *P* < 0.001), whereas gross H1 and/or P1 disease did not (*P* = 0.085).

**Table 2:** Findings at staging laparoscopy and subsequent curative-intent resection

	Patients (*n* of *N*)
SL-positive, overall	141 of 504 (28.0%)
**Pattern among SL-positive patients**	
CY1 only	90 of 141 (63.8%)
P1 only	1 of 141 (0.7%)
H1 only	15 of 141 (10.6%)
CY1 + P1	19 of 141 (13.5%)
CY1 + H1	8 of 141 (5.7%)
CY1 + P1 + H1	8 of 141 (5.7%)
**SL positivity by resectability category**	
R	56 of 263 (21.3%)
BR	35 of 121 (28.9%)
BR-PV	4 of 25 (16.0%)
BR-A	31 of 96 (32.3%)
UR-LA	50 of 120 (41.7%)
Curative-intent resection achieved (overall)	286 of 504 (56.7%)
Subsequent curative-intent resection among SL-positive patients	30 of 141 (21.3%)
**Subsequent curative-intent resection among SL-positive patients by resectability category**	
R	21 of 56 (37.5%)
BR	7 of 35 (20.0%)
BR-PV	1 of 4 (25.0%)
BR-A	6 of 31 (19.4%)
UR-LA	2 of 50 (4.0%)

Values are *n* of N (%). The six observed SL-positive patterns are mutually exclusive and together account for all 141 SL-positive patients; no patient had concurrent H1 and P1 without CY1. ‘Only’ indicates absence of the other two findings. Pattern percentages use all SL-positive patients as the denominator. SL positivity percentages use all patients in the corresponding resectability category. Curative-intent resection percentages use the full cohort or the SL-positive cohort, as applicable. SL, staging laparoscopy; CY1, positive peritoneal cytology; H1, liver metastasis; P1, macroscopic peritoneal metastasis; R, resectable; BR, borderline resectable, comprising BR-PV and BR-A; BR-PV, borderline resectable with portal vein involvement; BR-A, borderline resectable with arterial involvement; UR-LA, unresectable locally advanced.

Cytology was the dominant contributor to the yield. CY1 was present in 125 of 141 positive laparoscopies (88.7%), whereas gross liver and peritoneal metastases were identified in 31 patients (22.0%) and 28 patients (19.9%), respectively; overall, only 51 patients (10.1% of the whole cohort) had visually evident H1 and/or P1 disease. CY1-only disease was the most common pattern, occurring in 90 patients (63.8% of all positive laparoscopies), followed by CY1 + P1 in 19, H1 only in 15, CY1 + H1 in 8, CY1 + P1 + H1 in 8, and P1 only in 1 (*Table 2*). Therefore, the principal incremental value of SL in this cohort was the detection of occult peritoneal dissemination by cytology rather than visual identification of macroscopic metastases. No patient had concurrent H1 and P1 without CY1.

Across the detailed resectability categories, CY1-only disease occurred in 37 of 263 patients (14.1%) with resectable disease, 1 of 25 (4.0%) with BR-PV, 16 of 96 (16.7%) with BR-A, and 36 of 120 (30.0%) with UR-LA disease; gross H1 and/or P1 disease occurred in 19 of 263 (7.2%), 3 of 25 (12.0%), 15 of 96 (15.6%), and 14 of 120 (11.7%) patients, respectively (*Table S1*).

Curative-intent resection was ultimately achieved in 286 of 504 patients (56.7%) during the overall treatment course and in 30 of 141 SL-positive patients (21.3%) (*Table 2*). Among patients with positive SL findings, subsequent curative-intent resection was achieved in 21 of 56 patients (37.5%) with initially resectable disease, 7 of 35 patients (20.0%) with borderline resectable disease, and 2 of 50 patients (4.0%) with UR-LA disease (*Table 2*). This endpoint reflects the downstream treatment course only and should not be interpreted as evidence of a survival benefit after conversion surgery.

A positive SL result generally altered the immediate treatment sequence: gross H1 and/or P1 disease was managed as overt metastatic disease, whereas CY1-only disease was treated as occult metastatic (M1-equivalent) disease. Accordingly, subsequent curative-intent resection after a positive SL represents carefully selected downstream surgery following systemic therapy and multidisciplinary reassessment, rather than routine upfront resection for CY1 disease. The score-based risk-enrichment analysis is presented in *Fig. 2*.

**Figure 2:**
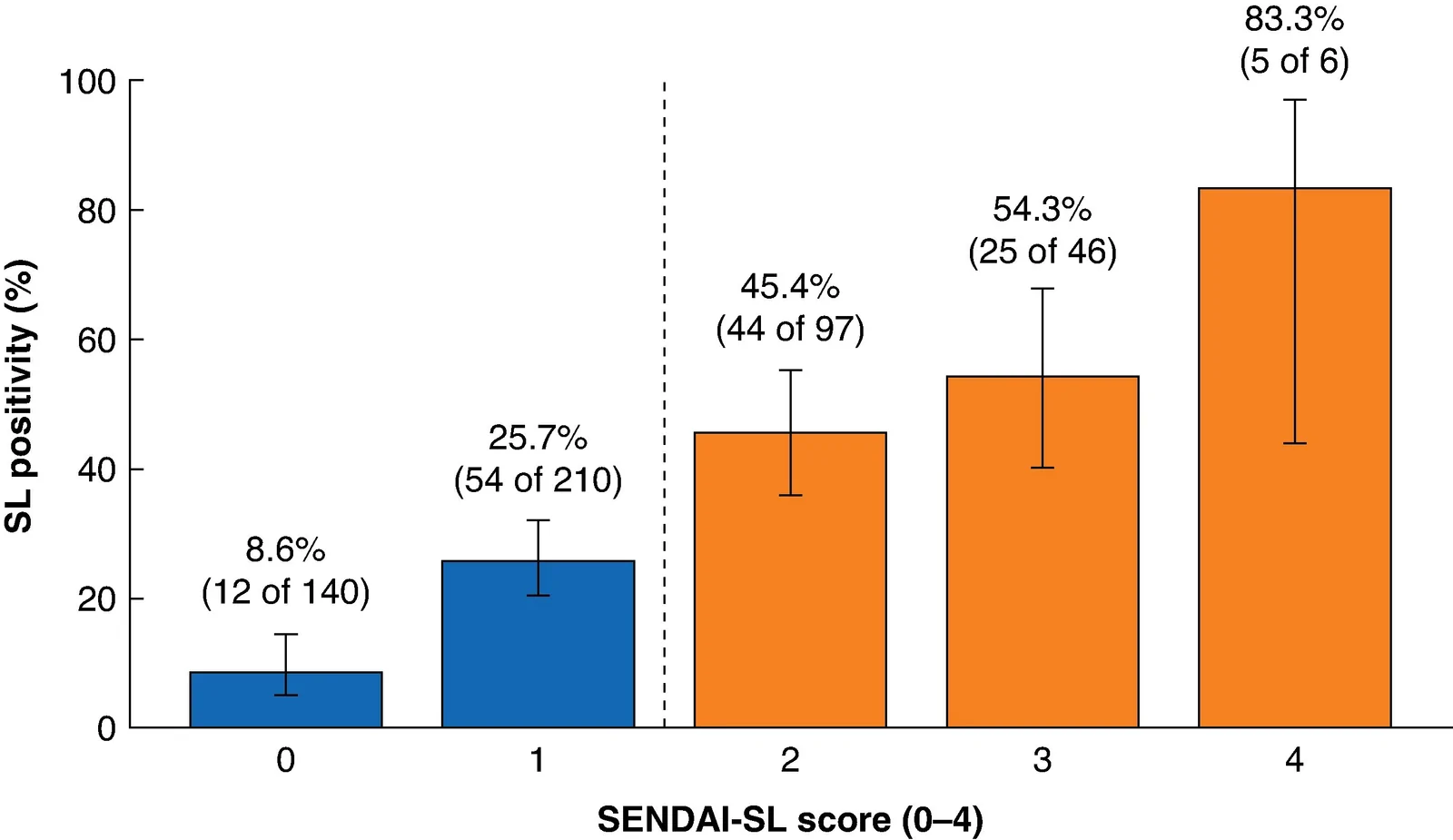
Observed staging laparoscopy positivity by SENDAI-SL score Bars show observed positivity, with error bars representing 95% confidence intervals in the analysis cohort with quantifiable CA19-9 values (499). The dashed line indicates the candidate high-priority threshold (≥ 2 points). The score is intended for risk enrichment and patient prioritization, not for omission of SL. SL, staging laparoscopy; CA19-9, carbohydrate antigen 19-9.

### Downstream treatment course and survival after positive SL

*Post hoc* analysis further characterized downstream outcomes among the 141 SL-positive patients with available follow-up. Subsequent conversion surgery (CS(+)), defined as curative-intent resection after the index positive SL, was performed in 30 patients (21.3%), after a median (i.q.r.) interval of 6.8 (5.5–9.0) months. The remaining 111 patients without subsequent curative-intent resection were classified as CS(−). Based on the initially assigned resectability category, the CS(+) group comprised 21 patients with resectable disease, 1 with BR-PV, 6 with BR-A, and 2 with UR-LA disease. Findings at the index SL were CY1-only disease in 25 patients, CY1 with gross H1 or P1 disease in 4, and isolated H1 in 1 (*Fig. 3a*).

**Figure 3:**
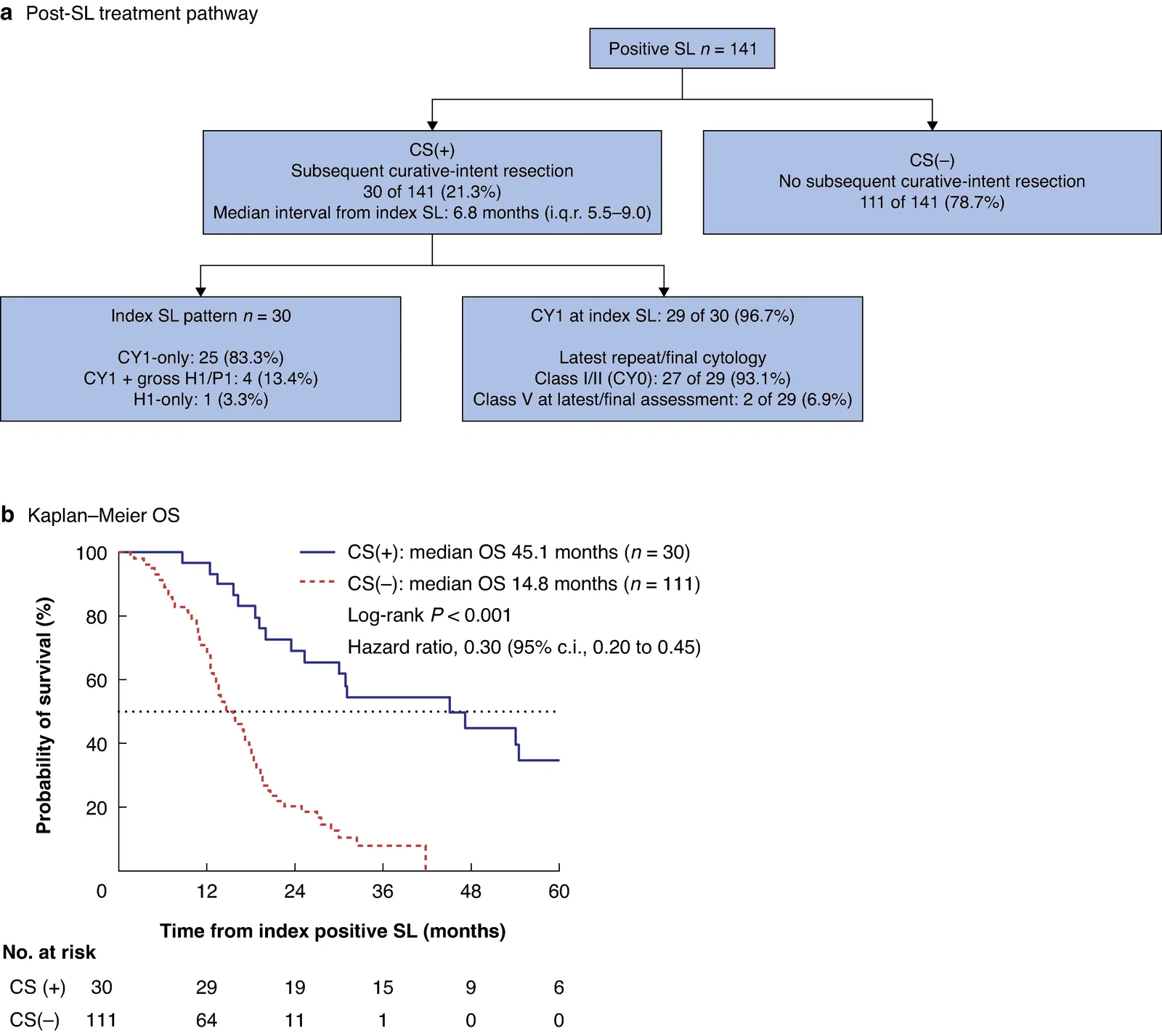
Downstream treatment course and survival after the index positive SL **a** Post-SL treatment pathway among 141 patients with positive SL findings. CS(+) denotes subsequent CS, defined as curative-intent resection after positive SL; CS(−) denotes no subsequent curative-intent resection. Among the 29 patients in the CS(+) group with CY1 at the index SL, the latest repeat or final cytology was Class I/II in 27 (93.1%) and Class V in 2 (6.9%). **b** Kaplan–Meier overall survival from the index positive SL stratified by subsequent CS status. Median OS was 45.1 months in the CS(+) group and 14.8 months in the CS(−) group (Mantel–Haenszel hazard ratio 0.30, 95% c.i. 0.20 to 0.45; log-rank *P* < 0.001). These exploratory findings are subject to selection and immortal-time bias and do not establish a causal benefit of conversion surgery. SL, staging laparoscopy; CS, conversion surgery; CY1, positive peritoneal cytology; OS, overall survival; c.i., confidence interval; i.q.r., interquartile range; H1, liver metastasis; P1, macroscopic peritoneal metastasis; CY0, negative peritoneal cytology.

Repeat or final cytology results were documented for all 29 patients in the CS(+) group with CY1 at the index SL. At the latest assessment, Class I/II cytology, consistent with conversion to CY0, was documented in 27 patients (93.1%), whereas Class V cytology was documented in 2 (6.9%). Nine of the 30 patients in the CS(+) group had received anticancer treatment before the index SL: gemcitabine plus S-1 in 5 (with subsequent chemoradiotherapy in 1), modified FOLFIRINOX (folinic acid, fluorouracil, irinotecan, oxaliplatin) in 2, gemcitabine plus nab-paclitaxel in 1, and S-1 plus radiotherapy in 1. After the index SL and before resection, 27 patients received anticancer treatment: 25 received a regimen or sequence incorporating gemcitabine plus nab-paclitaxel, 1 received modified FOLFIRINOX alone, and 1 received modified FOLFIRINOX followed by gemcitabine plus S-1. Resection margins were microscopically negative (R0) in 25 patients (83.3%) and microscopically positive (R1) in five (16.7%).

Median OS from the index positive SL was 45.1 months in the CS(+) group and 14.8 months in the CS(−) group (Mantel–Haenszel hazard ratio 0.30, 95% c.i. 0.20 to 0.45; log-rank *P* < 0.001) (*Fig. 3b*). All 30 patients in the CS(+) group had a valid postresection event or censoring intervals; recurrence or death occurred in 25, and the exploratory Kaplan–Meier median DFS from resection was 14.1 months. Because later resection was contingent on sustained disease control, preserved performance status, absence of newly evident dissemination, and survival until resection, this comparison is subject to selection and immortal-time bias and should be interpreted descriptively, not as evidence of a causal benefit from conversion surgery.

### Predictors of SL positivity and model performance

In the analysis cohort with quantifiable CA19-9 values (499), four factors available at the pre-SL decision point remained independently associated with SL positivity in the multivariable analysis (*Table 3*). Compared with resectable disease, UR-LA disease was associated with greater odds of SL positivity (adjusted odds ratio (aOR) 2.02, 95% c.i. 1.22 to 3.34). Body and/or tail location (aOR 2.84, 1.83 to 4.41), tumour size of at least 37 mm (aOR 2.61, 1.56 to 4.37), and CA19-9 of at least 180 U/ml (aOR 2.44, 1.55 to 3.83) were also independently associated with positivity. BR-A showed a numerical but non-significant association after adjustment, whereas BR-PV did not (*Table 3*).

**Table 3:** Logistic regression analysis of factors associated with staging laparoscopy positivity

	Univariable OR	Univariable *P*	Multivariable OR	Multivariable *P*
BR-PV *versus* R	0.69 (0.23, 2.09)	0.513	0.82 (0.25, 2.61)	0.731
BR-A *versus* R	1.67 (0.99, 2.83)	0.054	1.70 (0.97, 2.99)	0.064
UR-LA *versus* R	2.59 (1.62, 4.14)	< 0.001	2.02 (1.22, 3.34)	0.007
Body and/or tail *versus* head	2.63 (1.77, 3.93)	< 0.001	2.84 (1.83, 4.41)	< 0.001
Tumour size ≥ 37 mm	3.58 (2.21, 5.77)	< 0.001	2.61 (1.56, 4.37)	< 0.001
CA19-9 ≥ 180 U/ml	2.18 (1.45, 3.28)	< 0.001	2.44 (1.55, 3.83)	< 0.001

Values in parentheses are 95% confidence intervals. Univariable and multivariable analyses were restricted to the analysis cohort with quantifiable CA19-9 values (499); five patients with values below the assay's lower limit of quantification (< 2.0 U/ml) were excluded. The multivariable model included the originally assigned resectability category, tumour location, tumour size ≥ 37 mm, and CA19-9 ≥ 180 U/ml. OR, odds ratio; BR-PV, borderline resectable with portal vein involvement; R, resectable; BR-A, borderline resectable with arterial involvement; UR-LA, unresectable locally advanced; CA19-9, carbohydrate antigen 19-9. *P* values were calculated using two-sided Wald tests in univariable and multivariable logistic regression analyses.

The full model showed moderate discrimination, with an apparent AUC of 0.732 (*Fig. S1*). Bootstrap validation indicated modest optimism: the corrected AUC was 0.718, the corrected Brier score was 0.179, the calibration intercept was −0.071, and the calibration slope was 0.914 (*Table S2*). Apparent and optimism-corrected calibration curves showed close agreement between predicted and observed risks over most probability ranges (*Fig. S2*).

### SENDAI-SL score

These four factors were simplified into a one-point-per-variable SENDAI-SL score ranging from 0 to 4. The observed SL positivity rate increased monotonically across strata from 8.6% (12 of 140) at a score of 0 to 25.7% (54 of 210) at 1, 45.4% (44 of 97) at 2, 54.3% (25 of 46) at 3, and 83.3% (5 of 6) at 4 (*Fig. 2* and *Table S3*). When treated as an ordinal predictor, the simplified score yielded an AUC of 0.717, which was close to that of the parent model. Because the score-4 stratum comprised only six patients, its results should be interpreted cautiously.

Using a threshold of ≥ 2 points, 149 of 499 patients (29.9%) were classified as high priority for SL, and 74 of these patients (49.7%) had occult metastatic disease. The corresponding number needed to scope—defined as the number of SL procedures required to identify one patient with occult metastatic disease—was approximately 2.0 (*Table S3*). This threshold identified 74 of 140 (52.9%) positive laparoscopies in the analysis cohort. However, 66 of 350 patients (18.9%) with scores 0–1 remained SL-positive, representing 47.1% of positive laparoscopies in the analysis cohort; 43 had CY1-only disease, and 23 had gross H1 and/or P1 disease (*Table S4*). Lowering the threshold to ≥ 1 increased sensitivity to 91.4% but reduced the yield to 35.7%, whereas restricting priority to scores of ≥ 3 improved the yield to 57.7% but identified only 10.4% of patients and captured 21.4% of positive laparoscopies (*Table S3*).

Observed positivity generally increased with the score across resectability strata (*Fig. S3*). In simplified analyses combining BR-PV and BR-A, positivity in resectable disease increased from 5.4% at score 0 to 75.0% at score 3. In borderline resectable disease, positivity increased from 14.6% at score 0 to 52.4% at score 2, and in UR-LA disease from 27.6% at score 1 to 83.3% at score 4 (*Tables S5*, *S6*). At the candidate threshold of ≥ 2, positivity was 60.0% (21 of 35) in resectable disease, 47.8% (11 of 23) in borderline resectable disease, and 46.2% (42 of 91) in UR-LA disease. These resectability-stratified analyses should be interpreted as exploratory because several BR-PV and BR-A subgroups were small and the score-4 stratum comprised only six patients. Because the positivity rate at a score of 0 remained appreciable, the score should be regarded as a hypothesis-generating prioritization tool rather than an exclusion rule. A pragmatic prioritization framework based on these strata is presented in *Fig. S4*.

### Exploratory analysis of treatment before SL

Of the 504 patients, 145 (28.8%) had initiated anticancer treatment before SL. These patients represented a clinically selected subgroup who remained radiographically non-metastatic and eligible for SL after the preceding treatment course. Compared with patients who underwent upfront SL, they were younger (mean 66.9 *versus* 69.7 years), more often had UR-LA disease (33.1% *versus* 20.1%), and, among patients with available PET data, had a lower median SUVmax (4.6 *versus* 6.1), reflecting clinically non-random treatment sequencing. Among patients with quantifiable CA19-9 values, median CA19-9 was lower in the pre-SL treatment group than in the upfront-SL group (29.9 *versus* 88.7 U/ml) (*Table S7*). The tumour location was similar between groups, and the frequency of tumours of at least 37 mm did not differ significantly (*Table S7*).

Within this selected SL cohort, SL positivity was similar between patients with and without pre-SL treatment: 39 of 145 (26.9%) *versus* 102 of 359 (28.4%). The CY1, P1, and H1 rates were similar, and CY1-only remained the dominant positive pattern in both groups (64.1% and 63.7% of SL-positive patients, respectively) (*Table S8*). In the analysis cohort with quantifiable CA19-9 values (499), pre-SL treatment was not independently associated with SL positivity after adjustment for the same core covariables used in the main model (aOR 0.97, 95% c.i. 0.60 to 1.58) (*Table S9*). This null association should be interpreted in the context of patients who remained eligible for SL and should not be taken as evidence that treatment timing is biologically irrelevant. In the stratified models, body and/or tail location remained associated with positivity in both treatment strata, whereas CA19-9 levels of at least 180 U/ml appeared more informative after pre-SL treatment, although confidence intervals were wider (*Table S9*). Curative-intent resection was achieved in 92 of 145 treated patients (63.4%) and 194 of 359 untreated patients (54.0%), and, among those resected, the interval from SL to surgery was shorter after pre-SL treatment (median 23 *versus* 81 days) (*Table S7*).

## Discussion

In this consecutive single-centre cohort, SL upstaged more than one-quarter of patients with radiographically non-metastatic PDAC despite contemporary imaging. CY1-only disease, rather than macroscopic metastatic disease, was the principal source of upstaging, accounting for 63.8% of positive laparoscopies. Four routinely available factors—the initially assigned UR-LA category, body and/or tail tumour location, tumour size of at least 37 mm, and CA19-9 of at least 180 U/ml—were independently associated with SL positivity. The SENDAI-SL score should therefore be viewed as a pragmatic, hypothesis-generating prioritization tool rather than a validated basis for omitting SL.

The overall SL yield was at the upper end of the reported range and may reflect routine lavage cytology, inclusion of UR-LA disease, and tertiary-centre referral patterns^3–6^. In this series, cytology provided most of the additional staging yield, supporting lavage cytology rather than visual inspection alone when SL is undertaken to improve staging accuracy. The clinical utility of SL depends substantially on anatomical resectability. In patients planned for upfront surgery, particularly those with radiographically resectable disease or selected BR-PV tumours, detection of H1, P1, or CY1 disease at SL may avert non-beneficial laparotomy or venous resection. In contrast, in BR-A and UR-LA disease, systemic therapy is usually indicated irrespective of SL findings; in these settings, SL is more likely to inform prognosis, treatment intent, trial stratification, and subsequent decisions regarding conversion surgery or other local treatment. This distinction is consistent with recent international survey data showing substantial variation in SL use and timing, with selective use based on high-risk features being most common^7^. Accordingly, SL need not precede chemotherapy in every patient with BR-A or UR-LA disease if this would delay timely systemic therapy.

The predominance of CY1-only disease suggests that the principal contribution of SL was the detection of occult peritoneal dissemination. Positive peritoneal cytology has been repeatedly associated with early systemic failure and poor survival, even when macroscopic metastases are absent^15–17^. In this cohort, detection of CY1-only disease altered treatment intent and sequencing: CY1 was managed as occult metastatic (M1-equivalent) disease, immediate pancreatectomy was generally deferred, and systemic chemotherapy was prioritized. Previous observational studies^8–10^ have reported more favourable outcomes among selected patients who achieved cytological clearance after systemic therapy and subsequently underwent resection than among those who underwent surgery with persistent positive cytology. Among the 29 patients with CY1 at the index SL who subsequently underwent resection, 27 (93.1%) had Class I/II cytology at the latest repeat or final assessment. A positive SL finding did not invariably preclude subsequent resection in carefully selected responders; however, the longer observed OS in the CS(+) group is descriptive and does not establish a causal survival benefit of conversion surgery because of selection and immortal-time bias. Recurrence or death after resection remained common, and the Kaplan–Meier median DFS among the 30 patients in the CS(+) group was 14.1 months, underscoring the persistent systemic risk after resection following an initially positive SL.

The SENDAI-SL score was designed for clinical usability rather than maximal modelling complexity. Existing preoperative models^18^ for occult metastatic disease have incorporated broader variable sets; however, external validation^19^ has shown that predictive performance may decline across institutions. In contrast, the four retained factors are available at the multidisciplinary decision point when SL is considered, require no specialized postprocessing, and preserve most of the full model's discrimination. The full model showed moderate discrimination (apparent AUC 0.732; optimism-corrected AUC 0.718), whereas the simplified score had an apparent AUC of 0.717. The proposed thresholds should be interpreted with caution. The 37-mm tumour-size threshold was derived in the present cohort, whereas the CA19-9 threshold of 180 U/ml was adopted from a previous ROC-based staging-laparoscopy cohort. These dichotomized thresholds should not be regarded as biological boundaries. Reported CA19-9 thresholds, in particular, vary according to population, endpoint, biliary status, and treatment context^8–10,14,20^. The tumour-size component warrants particular caution after systemic therapy because residual soft tissue may represent viable tumour, desmoplastic stroma, inflammation, fibrosis, or treatment-related change, and radiological response does not necessarily reflect pathological response or true resectability^1,21^. Accordingly, the score combines the originally assigned resectability category and tumour location with tumour size and CA19-9 measured at the pre-SL assessment. It should be interpreted as an estimate of occult metastatic risk at the pre-SL assessment rather than as a measure of baseline tumour biology or a post-treatment criterion for resectability.

The principal limitation of a selective SL strategy is the potential to miss occult metastatic disease. At a threshold of ≥ 2 points, the score prioritized approximately 30% of patients and yielded nearly 50% positivity, but identified only 74 of 140 (52.9%) positive laparoscopies in the analysis cohort. Among patients below this threshold, 66 remained SL-positive, including 43 with CY1-only disease and 23 with gross H1 and/or P1 disease. Potential consequences include non-beneficial laparotomy or pancreatectomy, unnecessary vascular dissection, delayed systemic therapy, failure to identify M1-equivalent CY1 disease, and inappropriate local treatment. Because no clinically acceptable miss rate for occult CY1/H1/P1 disease has been established, the SENDAI-SL score should not be used to justify the routine omission of SL solely on the basis of a score below a specified threshold. Instead, its most appropriate role is to prioritize patients when resources for SL are limited, while ensuring that lower-scoring patients remain eligible for SL when clinical judgement, imaging findings, CA19-9 kinetics, or the planned operative strategy indicate potential benefit. Within the resectable subgroup, the candidate threshold of ≥ 2 identified only 35 of 259 patients (13.5%) but yielded a 60.0% positivity rate. However, it captured only 37.5% of positive resectable cases, and patients with a score of 0 still had a 5.4% positivity rate. These findings support selective prioritization before planned laparotomy rather than omission of SL in lower-scoring patients.

The optimal timing of SL remains unresolved. In patients proceeding directly to surgery, SL close to planned laparotomy may minimize interval progression. Conversely, in patients receiving induction therapy, SL may be performed either before treatment for baseline staging or after treatment to avoid futile exploration in those subsequently selected for resection or other local treatment. However, because the interval between the last chemotherapy cycle and SL, the reasons for treatment interruption, and interval progression before SL were not uniformly captured, prospective studies are warranted to define the optimal sequencing of SL. After adjustment, no independent association was found between treatment before SL and SL positivity; however, this should not be interpreted as evidence that sequencing is biologically neutral. Patients who developed radiographically evident metastases, clinical deterioration, or loss of local-treatment candidacy during chemotherapy would generally not proceed to SL and were therefore not represented in the treated SL subgroup. The analysis of pre-SL treatment is consequently conditional on remaining radiographically non-metastatic and clinically eligible for SL.

Future staging strategies should integrate anatomical, biological, molecular, and operative information rather than rely on a single modality. SL with cytology directly samples the peritoneal compartment, whereas plasma-based biomarkers may better reflect systemic disease. Emerging circulating tumour DNA approaches, including mutant KRAS detection in plasma and peritoneal fluid, have shown promise for identifying patients at increased risk of occult metastasis, metastatic progression, and poor survival^22^. These biomarkers may complement imaging, CA19-9 kinetics, cytology, and SL, but are not established substitutes for direct peritoneal assessment. In addition to metastatic staging, SL may facilitate local restaging by revealing unexpected local extension, adhesions, inflammatory peritoneal changes, or operative constraints that influence the feasibility or strategy of pancreatectomy. Because such observations were not systematically recorded in the present study, future prospective SL registries should capture both metastatic and local restaging endpoints.

This study has several limitations. First, this retrospective, single-centre study spanned more than 12 years, during which imaging, diagnostic work-up, systemic therapy, operative strategies, and indications for SL evolved. SL was selective rather than protocol-mandated, creating potential selection bias. Second, the score was developed and evaluated in the same cohort; bootstrap optimism correction cannot substitute for external validation. The score-4 category included only six patients, several resectability-by-score cells were small, the 37-mm threshold was data-derived, and dichotomization of tumour size and CA19-9 entailed information loss and potential optimism. The 180-U/ml CA19-9 threshold was adopted from the literature but is not universally validated. Third, the model combined chemotherapy-naïve and selected post-treatment patients at the pre-SL decision point, although post-treatment tumour size and CA19-9 are not biologically equivalent to baseline values. CA19-9 kinetics, treatment-specific thresholds, and the interval from the last treatment dose to SL were not uniformly available. The treatment analysis was also affected by survivorship and selection bias because patients who progressed before SL were not captured. Fourth, formal decision curve analysis was not performed, and an acceptable miss rate for occult CY1/H1/P1 disease was not defined. Fifth, post-SL treatment, latest repeat or final cytology, OS, and DFS were analysed *post hoc*; local restaging findings were not systematically recorded. The association between later resection and survival therefore must not be interpreted as a causal effect of conversion surgery. Strengths include a large consecutive contemporary cohort, routine lavage cytology, inclusion of the full resectability spectrum, detailed characterization of positive findings, and bootstrap assessment of a clinically simple model.

In conclusion, in patients with radiographically non-metastatic PDAC, SL upstaged more than one-quarter of cases, largely by identifying cytology-only micrometastatic disease. The SENDAI-SL score may help prioritize SL when resources are limited; however, it should not be used to categorically omit SL and requires external validation.

## Supplementary Material

zrag176_Supplementary_Data

## Data Availability

De-identified data are not publicly available because of patient confidentiality and institutional policy but may be available from the corresponding author upon reasonable request, subject to institutional approval.
