# Supplementary material for: Staging laparoscopy for radiographically non-metastatic pancreatic ductal adenocarcinoma: cytology-driven upstaging and the SENDAI-SL risk-enrichment score
Source: BJS Open. 2026 Nov 4;10(6):zrag176. doi: 10.1093/bjsopen/zrag176 (PMC13733517; doi:10.1093/bjsopen/zrag176)
Supplement: Supplementary file 1 [file zrag176_supplementary_data.docx]

Staging laparoscopy for radiographically non-metastatic pancreatic ductal adenocarcinoma: cytology-driven upstaging and the SENDAI-SL risk-enrichment score

Daisuke Douchi^1^, Shingo Yoshimachi^1^, Naoki Rikiyama^1^, Mika Ando^1^, Yuichiro Umino^1^, Hideaki Sato^1^, Mitsuhiro Shimura^1^, Koetsu Inoue^1^, Masahiro Iseki^1^, Shuichi Aoki^1^, Takayuki Miura^1^, Shimpei Maeda^1^, Masaharu Ishida^1^, Masamichi Mizuma^1^, Atsushi Masamune^2^, Michiaki Unno^1^

^1^Department of Surgery, Tohoku University Graduate School of Medicine, Sendai, Japan

^2^Division of Gastroenterology, Tohoku University Graduate School of Medicine, Sendai, Japan

**Correspondence to:** Daisuke Douchi

Department of Surgery, Tohoku University Graduate School of Medicine

1-1 Seiryo-machi, Aoba-ku, Sendai, Miyagi 980-8574, Japan

Tel.: +81-22-717-7201

Fax: +81-22-717-7209

E-mail: daisuke.douchi.e6@tohoku.ac.jp

**ORCID:** 0000-0003-4582-6589

**Supplementary Materials - Index**

| **Supplementary Figures and Tables** |  |
| --- | --- |
| Supplementary Figure S1 | *page 2* |
| Supplementary Figure S2 | *page 3* |
| Supplementary Figure S3 | *page 4* |
| Supplementary Figure S4 | *page 5* |
| Supplementary Table S1 | *page 6* |
| Supplementary Table S2 | *page 6* |
| Supplementary Table S3 | *page 7* |
| Supplementary Table S4 | *page 8* |
| Supplementary Table S5 | *page 8* |
| Supplementary Table S6 | *page 8* |
| Supplementary Table S7 | *page 9* |
| Supplementary Table S8 | *page 10* |
| Supplementary Table S9 | *page 11* |

**Supplementary Figures**

**Supplementary Figure S1. Receiver operating characteristic curve for the multivariable model predicting staging laparoscopy positivity**

The model included resectability category, body/tail location, tumour size ≥37 mm, and CA19-9 ≥180 U/mL in the analysis cohort of 499 patients with quantifiable CA19-9 values; five patients with values below the assay's lower limit of quantification (<2.0 U/mL) were excluded. AUC: area under the receiver operating characteristic curve; CA19-9: carbohydrate antigen 19-9.

**Supplementary Figure S2. Apparent and bootstrap optimism-corrected calibration of the multivariable model**

The dashed diagonal line indicates ideal calibration. The blue curve represents apparent calibration, and the orange curve represents bootstrap optimism-corrected calibration based on 2 000 resamples; circles indicate grouped observed risk with Wilson 95% confidence intervals. The model included resectability category, body/tail location, tumour size ≥37 mm, and CA19-9 ≥180 U/mL in the analysis cohort with quantifiable CA19-9 values (n = 499); five patients with values below the assay's lower limit of quantification (<2.0 U/mL) were excluded. CI: confidence interval; LOWESS: locally weighted scatterplot smoothing.

**Supplementary Figure S3. Staging laparoscopy positivity by resectability category and SENDAI-SL score**

Panel a represents detailed categories (resectable, BR-PV, BR-A, and UR-LA). Panel b presents simplified categories, with BR-PV and BR-A combined as borderline resectable. Each cell shows SL-positive patients as n of N (%). Grey cells indicate non-applicable combinations or absence of patients; cells with fewer than five patients should be interpreted cautiously. BR: borderline resectable; BR-A: borderline resectable with arterial involvement; BR-PV: borderline resectable with portal vein involvement; R: resectable; SL: staging laparoscopy; UR-LA: unresectable locally advanced.

**Supplementary Figure S4. Proposed prioritisation framework for staging laparoscopy using the SENDAI-SL score**

The score assigns 1 point each for originally assigned UR-LA status, body/tail location, tumour size ≥37 mm, and CA19-9 ≥180 U/mL. Tumour size and CA19-9 should be derived from the same pre-SL decision point; in patients assessed after induction chemotherapy, the most recent post-treatment, pre-SL values should be used, and baseline and post-treatment values should not be combined. A threshold of ≥2 points identifies a high-priority group with 49.7% positivity (74 of 149) in the analysis cohort with quantifiable CA19-9 values (n = 499). The framework is intended to prioritise SL when diagnostic or operative capacity is constrained; it is not intended to mandate SL before chemotherapy in all patients. In BR-A and UR-LA disease, timely systemic therapy should not be delayed solely to perform SL. BR: borderline resectable; BR-A: borderline resectable with arterial involvement; BR-PV: borderline resectable with portal vein involvement; CA19-9: carbohydrate antigen 19-9; PDAC: pancreatic ductal adenocarcinoma; R: resectable; SL: staging laparoscopy; UR-LA: unresectable locally advanced.

**Supplementary Tables**

**Supplementary Table S1. Staging laparoscopy findings stratified by detailed resectability category**

| **Resectability** | **n** | **SL-positive,**  **n (%)** | **CY1, n (%)** | **CY1-only, n (%)** | **Gross H1/P1, n (%)** | **H1, n (%)** | **P1, n (%)** |
| --- | --- | --- | --- | --- | --- | --- | --- |
| Overall | 504 | 141 (28.0) | 125 (24.8) | 90 (17.9) | 51 (10.1) | 31 (6.2) | 28 (5.6) |
| R | 263 | 56 (21.3) | 50 (19.0) | 37 (14.1) | 19 (7.2) | 14 (5.3) | 8 (3.0) |
| BR-PV | 25 | 4 (16.0) | 3 (12.0) | 1 (4.0) | 3 (12.0) | 2 (8.0) | 1 (4.0) |
| BR-A | 96 | 31 (32.3) | 26 (27.1) | 16 (16.7) | 15 (15.6) | 8 (8.3) | 11 (11.5) |
| UR-LA | 120 | 50 (41.7) | 46 (38.3) | 36 (30.0) | 14 (11.7) | 7 (5.8) | 8 (6.7) |
| P value† |  | < 0.001 | < 0.001 | < 0.001 | 0.085 |  |  |

Values are n (%) unless otherwise stated. Percentages are calculated within each resectability stratum. SL positivity was defined as H1 and/or P1 and/or CY1. CY1 includes all patients with positive lavage cytology. CY1-only was defined as CY1 without H1 or P1. Gross H1/P1 was defined as H1 and/or P1 regardless of CY1 status.

† P values were calculated across the four detailed resectability categories using the Fisher–Freeman–Halton exact test for 4 × 2 tables. H1 and P1 were reported descriptively and were not included in the prespecified resectability-stratified comparisons.

Abbreviations: BR-A, borderline resectable with arterial involvement; BR-PV, borderline resectable with portal vein involvement; CY1, positive peritoneal cytology; H1, liver metastasis; P1, macroscopic peritoneal metastasis; R, resectable; SL, staging laparoscopy; UR-LA, unresectable locally advanced.

**Supplementary Table S2. Bootstrap internal validation metrics for the multivariable model predicting staging laparoscopy positivity**

| **Metric** | **Apparent estimate** | **Mean optimism** | **Optimism-corrected estimate** |
| --- | --- | --- | --- |
| AUC | 0.732 | 0.015 | 0.718 |
| Brier score | 0.174 | 0.005 | 0.179 |
| Calibration intercept | 0.000 | 0.071 | −0.071 |
| Calibration slope | 1.000 | 0.086 | 0.914 |

Apparent calibration intercept and slope are 0 and 1 by construction.

Bootstrap internal validation used 2 000 resamples in the analysis cohort with quantifiable CA19-9 values (n = 499). For calibration intercept and slope, mean optimism denotes the average departure from the ideal values (0 and 1, respectively). AUC: area under the receiver operating characteristic curve; CA19-9: carbohydrate antigen 19-9.

**Supplementary Table S3. Performance of the SENDAI-SL score and candidate prioritisation thresholds**

| **Score** | **n** | **SL-positive, n (%)** | **95% CI, % (Wilson)** |
| --- | --- | --- | --- |
| 0 | 140 | 12 (8.6) | 5.0–14.4 |
| 1 | 210 | 54 (25.7) | 20.3–32.0 |
| 2 | 97 | 44 (45.4) | 35.8–55.3 |
| 3 | 46 | 25 (54.3) | 40.2–67.8 |
| 4 | 6 | 5 (83.3) | 43.6–97.0 |

**Candidate cutoffs for preferential allocation of SL**

| **Cutoff (score ≥)** | **n (%)** | **SL-positive, n (%)** | **Sensitivity, %** | **NNS** |
| --- | --- | --- | --- | --- |
| 1 | 359 (71.9) | 128 (35.7) | 91.4 | 2.80 |
| **2** | **149 (29.9)** | **74 (49.7)** | **52.9** | **2.01** |
| 3 | 52 (10.4) | 30 (57.7) | 21.4 | 1.73 |
| 4 | 6 (1.2) | 5 (83.3) | 3.6 | 1.20 |

Candidate high-priority cutoff for preferential allocation of SL: score ≥2 (risk enrichment only; not an exclusion rule).

Score = originally assigned UR-LA status + body/tail location + tumour size ≥37 mm + CA19-9 ≥180 U/mL (1 point each).

Sensitivity was calculated among SL-positive patients (n = 140) in the analysis cohort with quantifiable CA19-9 values (n = 499). CI: confidence interval; NNS: number needed to scope; SL: staging laparoscopy.

If used as a sole allocation rule, score ≥2 would not prioritise 350 of 499 patients and would miss 66 of 140 positive laparoscopies; therefore, low-score patients should not be excluded from SL solely on the basis of the score.

Abbreviations: CA19-9, carbohydrate antigen 19-9; UR-LA, unresectable locally advanced.

**Supplementary Table S4. Occult metastatic pattern by SENDAI-SL score group and missed positives below the ≥2 threshold**

| **Score group** | **n** | **SL-positive, n (%)** | **CY1-only among positives, n (%)** | **Gross H1/P1 among positives, n (%)** | **Missed positives if using ≥2 cutoff, n** |
| --- | --- | --- | --- | --- | --- |
| Score 0 | 140 | 12 (8.6) | 4 (33.3) | 8 (66.7) | 12 |
| Score 1 | 210 | 54 (25.7) | 39 (72.2) | 15 (27.8) | 54 |
| Score ≥2 | 149 | 74 (49.7) | 46 (62.2) | 28 (37.8) | — |
| Score 0–1 (below cutoff) | 350 | 66 (18.9) | 43 (65.2) | 23 (34.8) | 66 |

The score table was calculated in the analysis cohort with quantifiable CA19-9 values (n = 499), matching Supplementary Table 3. SL positivity was defined as H1 and/or P1 and/or CY1. CY1-only among positives was defined as CY1 without H1 or P1 among SL-positive patients. Gross H1/P1 among positives was defined as H1 and/or P1 among SL-positive patients, regardless of CY1 status. Percentages for CY1-only and gross H1/P1 are calculated among SL-positive patients within each score group. SL-positive cases among patients with scores of 0 or 1 would be missed if SL were allocated only to patients with a score ≥2.

Abbreviations: CA19-9, carbohydrate antigen 19-9; CY1, positive peritoneal cytology; H1, liver metastasis; P1, macroscopic peritoneal metastasis; SL, staging laparoscopy.

**Supplementary Table S5. Staging laparoscopy positivity stratified by detailed resectability category and SENDAI-SL score**

| **Resectability** | **0** | **1** | **2** | **3** | **4** |
| --- | --- | --- | --- | --- | --- |
| R | 5 of 92 (5.4%) | 30 of 132 (22.7%) | 15 of 27 (55.6%) | 6 of 8 (75.0%) | NA |
| BR-PV | 1 of 11 (9.1%) | 3 of 11 (27.3%) | 0 of 3† (0.0%) | — | NA |
| BR-A | 6 of 37 (16.2%) | 13 of 38 (34.2%) | 11 of 18 (61.1%) | 0 of 2† (0.0%) | NA |
| UR-LA | NA | 8 of 29 (27.6%) | 18 of 49 (36.7%) | 19 of 36 (52.8%) | 5 of 6 (83.3%) |

The analysis included 499 patients with quantifiable CA19-9 values. NA = not applicable by definition (UR-LA requires ≥1 point; score 4 requires UR-LA).

— indicates no patients. † Cells with n < 5 should be interpreted cautiously.

Abbreviations: BR-A, borderline resectable with arterial involvement; BR-PV, borderline resectable with portal vein involvement; CA19-9, carbohydrate antigen 19-9; R, resectable; SL, staging laparoscopy; UR-LA, unresectable locally advanced.

**Supplementary Table S6. Staging laparoscopy positivity stratified by simplified resectability category and SENDAI-SL score**

| **Resectability** | **0** | **1** | **2** | **3** | **4** |
| --- | --- | --- | --- | --- | --- |
| R | 5 of 92 (5.4%) | 30 of 132 (22.7%) | 15 of 27 (55.6%) | 6 of 8 (75.0%) | NA |
| BR | 7 of 48 (14.6%) | 16 of 49 (32.7%) | 11 of 21 (52.4%) | 0 of 2† (0.0%) | NA |
| UR-LA | NA | 8 of 29 (27.6%) | 18 of 49 (36.7%) | 19 of 36 (52.8%) | 5 of 6 (83.3%) |

The analysis included 499 patients with quantifiable CA19-9 values. BR combines BR-PV and BR-A. NA = not applicable by definition (UR-LA requires ≥1 point; score 4 requires UR-LA).

† Cells with n < 5 should be interpreted cautiously.

Abbreviations: BR, borderline resectable; BR-A, borderline resectable with arterial involvement; BR-PV, borderline resectable with portal vein involvement; CA19-9, carbohydrate antigen 19-9; R, resectable; SL, staging laparoscopy; UR-LA, unresectable locally advanced.

**Supplementary Table S7.
Pre-SL characteristics and selected treatment-course outcomes according to treatment before staging laparoscopy**

| **Characteristic** | **Overall (n = 504)** | **No pre-SL treatment (n = 359)** | **Pre-SL treatment (n = 145)** | **P value** |
| --- | --- | --- | --- | --- |
| Age, years, mean (s.d.) | 68.9 (9.1) | 69.7 (9.1) | 66.9 (8.6) | 0.001 |
| Male sex | 290 (57.5) | 206 (57.4) | 84 (57.9) | 0.910 |
| Resectability |  |  |  | < 0.001 |
| R | 263 (52.2) | 208 (57.9) | 55 (37.9) |  |
| BR | 121 (24.0) | 79 (22.0) | 42 (29.0) |  |
| BR-PV | 25 (5.0) | 15 (4.2) | 10 (6.9) |  |
| BR-A | 96 (19.0) | 64 (17.8) | 32 (22.1) |  |
| UR-LA | 120 (23.8) | 72 (20.1) | 48 (33.1) |  |
| Tumour location |  |  |  | 0.817 |
| Head | 296 (58.7) | 212 (59.1) | 84 (57.9) |  |
| Body/tail | 208 (41.3) | 147 (40.9) | 61 (42.1) |  |
| Tumour size, mm, median (i.q.r.) | 25.0 (18.0–32.0) | 25.0 (19.0–32.5) | 24.0 (16.0–30.0) | 0.017 |
| ≥37 mm | 87 (17.3) | 68 (18.9) | 19 (13.1) | 0.116 |
| CA19-9, U/mL, median (i.q.r.)† | 64.5 (16.1–247.5) | 88.7 (18.3–298.6) | 29.9 (12.6–122.1) | < 0.001 |
| ≥180 U/mL | 155 (31.1) | 125 (35.3) | 30 (20.7) | 0.002 |
| CEA, ng/mL, median (i.q.r.) | 3.1 (2.0–5.1) | 3.1 (2.0–5.1) | 3.0 (2.1–5.4) | 0.883 |
| CA125, U/mL, median (i.q.r.)‡ | 13.5 (9.9–21.1) | 14.0 (10.1–21.0) | 12.4 (9.5–22.1) | 0.832 |
| PET SUVmax, median (i.q.r.)§ | 5.6 (3.8–8.1) | 6.1 (4.1–8.4) | 4.6 (3.1–6.1) | < 0.001 |
| SL findings |  |  |  |  |
| CY1 | 125 (24.8) | 92 (25.6) | 33 (22.8) | 0.500 |
| P1 | 28 (5.6) | 20 (5.6) | 8 (5.5) | 0.981 |
| H1 | 31 (6.2) | 23 (6.4) | 8 (5.5) | 0.707 |
| SL-positive (H1/P1/CY1) | 141 (28.0) | 102 (28.4) | 39 (26.9) | 0.731 |
| Curative-intent resection achieved | 286 (56.7) | 194 (54.0) | 92 (63.4) | 0.054 |
| Time from SL to curative-intent resection, days, median (i.q.r.)¶ | 77.0 (23.0–98.0) | 81.0 (69.0–100.0) | 23.0 (15.0–85.0) | < 0.001 |

Values are n (%) unless otherwise stated. † CA19-9 was quantifiable in 499 patients (354 without and 145 with pre-SL treatment); five patients with values below the assay's lower limit of quantification (<2.0 U/mL) were excluded from CA19-9 analyses. ‡ CA125 was available in 291 patients. § PET SUVmax was available in 423 patients.

¶ Time from SL to curative-intent resection was calculated among patients who underwent curative-intent resection with available dates and non-negative intervals (n = 272).

P values were calculated using Welch's t-test for age, the Mann–Whitney U test for other continuous variables, and Pearson's χ² test for categorical variables. The resectability P value compares the four detailed categories (R, BR-PV, BR-A, and UR-LA).

Abbreviations: BR, borderline resectable; BR-A, borderline resectable with arterial involvement; BR-PV, borderline resectable with portal vein involvement; CA19-9, carbohydrate antigen 19-9; CA125, cancer antigen 125; CEA, carcinoembryonic antigen; CY1, positive peritoneal cytology; H1, liver metastasis; i.q.r., interquartile range; P1, macroscopic peritoneal metastasis; PET, positron emission tomography; R, resectable; s.d., standard deviation; SL, staging laparoscopy; SUVmax, maximum standardised uptake value; UR-LA, unresectable locally advanced.

**Supplementary Table S8. Staging laparoscopy findings and curative-intent resection according to treatment before staging laparoscopy**

| **Variable** | **No pre-SL treatment (n = 359)** | **Pre-SL treatment (n = 145)** | **P value** |
| --- | --- | --- | --- |
| SL-positive (H1/P1/CY1) | 102 (28.4) | 39 (26.9) | 0.731 |
| CY1 | 92 (25.6) | 33 (22.8) | 0.500 |
| P1 | 20 (5.6) | 8 (5.5) | 0.981 |
| H1 | 23 (6.4) | 8 (5.5) | 0.707 |
| Pattern among SL-positive |  |  | 0.661 |
| CY1 only | 65 (63.7) | 25 (64.1) |  |
| CY1 + P1 | 14 (13.7) | 5 (12.8) |  |
| CY1 + H1 | 7 (6.9) | 1 (2.6) |  |
| CY1 + P1 + H1 | 6 (5.9) | 2 (5.1) |  |
| H1 only | 10 (9.8) | 5 (12.8) |  |
| P1 only | 0 (0.0) | 1 (2.6) |  |
| P1 + H1 | 0 (0.0) | 0 (0.0) |  |
| Curative-intent resection achieved (overall) | 194 (54.0) | 92 (63.4) | 0.054 |
| Curative-intent resection achieved (among SL-positive) | 21 (20.6) | 9 (23.1) | 0.747 |

Values are n (%) unless otherwise stated. P values were calculated using Pearson's χ² test for binary outcomes and the Fisher–Freeman–Halton exact test for the pattern distribution; the all-zero P1 + H1 category was excluded from the pattern test.

Abbreviations: CY1, positive peritoneal cytology; H1, liver metastasis; P1, macroscopic peritoneal metastasis; SL, staging laparoscopy.

**Supplementary Table S9. Adjusted associations with staging laparoscopy positivity overall and according to treatment before staging laparoscopy**

| **Variable** | **Overall aOR (95% CI)** | **P value** | **No pre-SL treatment aOR (95% CI)** | **P value** | **Pre-SL treatment aOR (95% CI)** | **P value** |
| --- | --- | --- | --- | --- | --- | --- |
| Pre-SL treatment (Yes versus No) | 0.97 (0.60–1.58) | 0.916 | — | — | — | — |
| BR-PV versus R | 0.82 (0.25–2.64) | 0.739 | 1.10 (0.28–4.28) | 0.891 | 0.50 (0.05–5.16) | 0.560 |
| BR-A versus R | 1.71 (0.97–3.01) | 0.064 | 1.41 (0.71–2.78) | 0.324 | 2.50 (0.87–7.20) | 0.091 |
| UR-LA versus R | 2.03 (1.21–3.40) | 0.007 | 2.44 (1.30–4.56) | 0.005 | 1.55 (0.59–4.06) | 0.372 |
| Body/tail versus head | 2.84 (1.83–4.41) | < 0.001 | 2.98 (1.76–5.02) | < 0.001 | 2.85 (1.21–6.69) | 0.016 |
| Tumour size ≥37 mm | 2.60 (1.55–4.37) | < 0.001 | 2.99 (1.64–5.43) | < 0.001 | 1.78 (0.60–5.27) | 0.300 |
| CA19-9 ≥180 U/mL | 2.43 (1.54–3.83) | < 0.001 | 2.18 (1.28–3.71) | 0.004 | 3.30 (1.31–8.29) | 0.011 |

The overall model included the analysis cohort with quantifiable CA19-9 values (n = 499). Treatment-stratified models included 354 of 359 patients without pre-SL treatment (five excluded because CA19-9 was below the lower limit of quantification) and all 145 patients with pre-SL treatment. Resectability was analysed according to the originally assigned category; tumour location was fixed, and tumour size and CA19-9 were measured at the pre-SL decision point. Because treatment sequencing was non-random, the stratified estimates are exploratory. aOR: adjusted odds ratio; BR-A: borderline resectable with arterial involvement; BR-PV: borderline resectable with portal vein involvement; CA19-9: carbohydrate antigen 19-9; CI: confidence interval; R: resectable; SL: staging laparoscopy; UR-LA: unresectable locally advanced.
